# Nucleic Acid Sensing by Toll-Like Receptors in the Endosomal Compartment

**DOI:** 10.3389/fimmu.2022.941931

**Published:** 2022-06-23

**Authors:** Kensuke Miyake, Takuma Shibata, Ryutaro Fukui, Ryota Sato, Shin-Ichiroh Saitoh, Yusuke Murakami

**Affiliations:** ^1^ Division of Innate Immunity, Department of Microbiology and Immunology, The Institute of Medical Science, The University of Tokyo, Minato-ku, Japan; ^2^ Faculty of Pharmacy, Department of Pharmaceutical Sciences and Research Institute of Pharmaceutical Sciences, Musashino University, Tokyo, Japan

**Keywords:** nuclease, nucleoside, autoimmune disease, endosome, toll-like receptor

## Abstract

Toll-like receptors (TLRs) respond to pathogen constituents, such as microbial lipids and nucleic acids (NAs). TLRs recognize NAs in endosomal compartments. Structural and functional studies have shown that recognition of NAs by TLRs depends on NA processing by RNases and DNases. DNase II-dependent DNA degradation is required for TLR9 responses to single-stranded DNAs, whereas RNase T2-dependent RNA degradation enables TLR7 and TLR8 to respond to nucleosides and oligoribonucleotides. In contrast, RNases and DNases negatively regulate TLR responses by degrading their ligands. RNase T2 negatively regulates TLR3 responses to degrading the TLR3 ligand double-stranded RNAs. Therefore, NA metabolism in the endosomal compartments affects the endosomal TLR responses. Dysregulation of NA metabolism in the endosomal compartment drives the TLR-dependent pathologies in human diseases.

## 1 Introduction

The Toll family of receptors are expressed in innate immune cells, such as macrophages and dendritic cells (DCs), and respond to pathogen components to activate defense responses during bacterial and viral infections. Nucleic acids (NAs) are sensed by a subfamily of toll-like receptors (TLRs) including TLR3, TLR7, TLR8, TLR9, and TLR13. These NA-sensing TLRs are localized in the endosomal compartment to prevent hazardous autoimmune responses (1). NA degradation in the endosomal compartment negatively regulates TLR responses to self-derived NAs. However, evidence for another reason of TLR localization to the endosomal compartment is emerging. Structural and functional studies have shown that NA-sensing TLRs sense NA-degradation products, such as oligonucleotides and nucleosides (2–4), demonstrating that NA degradation generates TLR ligands. NA metabolism in the endosomal compartment is considered a positive and negative regulator of NA-sensing TLRs.

The endosomal compartment affects downstream signaling as well as NA sensing. TLRs activate two signaling pathways: proinflammatory signals activating NF-κB transcription factors and type I interferon signals activating transcription factors called interferon regulatory factors (IRFs) (5, 6). Both signals are activated in a mutually exclusive manner with the former preceding the latter pathway. Delayed activation of IRFs is ascribed to the requirement of endosomal trafficking for IRF activation by TLRs. Therefore, endosomal trafficking serves as a switch to change TLR responses from proinflammatory to type I interferon (IFN) responses.

Constitutive activation of NA-sensing TLRs causes inflammatory diseases. Constitutive TLR activation is caused by alteration in NA metabolism, the endosomal compartment, or downstream signaling. These inflammatory diseases reveal molecular and cellular mechanisms by which endosomal TLRs are controlled by the endosomal compartment (1, 7).

Here, we provide an overview of recent progress in our understanding of the mechanisms by which endosomal TLR responses are controlled and the diseases caused by dysregulation of these controlling mechanisms.

### 1.1 Nucleic Acid Recognition by TLRs in the Endosomal Compartment

#### 1.1.1 TLR3

TLR3 responds to double-stranded RNAs (dsRNAs) longer than 40–50 bp (8); this length is required to interact with a pair of TLR3 molecules and induce their dimerization. However, it remains unclear whether longer dsRNAs induce stronger TLR3 responses. TLR3 is expressed not only in the innate immune cells, such as macrophages and dendritic cells, but also in non-immune cells, such as neurons and keratinocytes. Broad expression enables TLR3 to serve as a sentinel protein in non-immune cells. For example, loss-of-function mutations in the genes required for TLR3-dependent type I IFN responses increase susceptibility to herpes encephalitis (9). Because neurons express only a limited set of pathogen sensors, TLR3 expressed in neurons plays an indispensable role in the control of herpes virus infection. TLR3 is probably activated by dsRNAs of virus origin, of which expression increase during viral infection. TLR3 also responds to self-derived RNAs during tissue damage. In keratinocytes, TLR3 responds to self-derived U1 RNA released from UV-irradiated cells to promote tissue repair (10). In contrast, TLR3 plays a pathologic role in radiation-induced gastrointestinal syndrome (11). TLR3 expressed in intestinal crypt cells responds to dsRNAs released p53-dependently from irradiated cells. Expression of TLR3 in crypt cells causes cell death and exacerbates radiation-induced gastrointestinal syndromes. Broad expression and responses to self-derived dsRNAs allow TLR3 to serve as a sensor not only for viruses but also for various tissue damage.

#### 1.1.2 TLR7, TLR8, and TLR13

TLR7 and TLR8 are known to respond to single-stranded RNAs (ssRNAs), but their structural and functional analyses have shown that these TLRs bind to nucleosides and oligoribonucleotides (2–4). TLR7 is activated by guanosine or deoxyguanosine along with oligoribonucleotides, whereas TLR8 responds to uridine and oligoribonucleotides. Nucleosides and oligonucleotides synergistically activate both TLRs because oligoribonucleotides enhance TLR7/8 affinity to nucleosides. TLR7 and TLR8, therefore, respond to RNA degradation products generated in the endosomal compartment. This is a strong reason for the localization of TLR7 and TLR8 in the endosomal compartment. In mice, TLR8 is not active, but TLR13 serves as a *bona fide* ssRNA sensor. TLR13 binds directly to bacterial 23S ribosomal RNA in a sequence-specific manner (12, 13).

#### 1.1.3 TLR9

TLR9 responds to single-stranded DNAs (ssDNAs). Because TLR9 has two binding sites, ssDNA fragment binds to a pair of TLR9 molecules, leading to the formation of a TLR9 dimer with two ssDNA fragments (14). Because cell surface expression of TLR9 drives systemic inflammation (15), ssDNA fragments may be present in the extracellular space as well as in the endosomal compartment.

### 1.2 Effect of Nucleic Acid Metabolism on Endosomal TLR Responses

#### 1.2.1 DNase I and DNase I-Like 3

**Figure 1:**
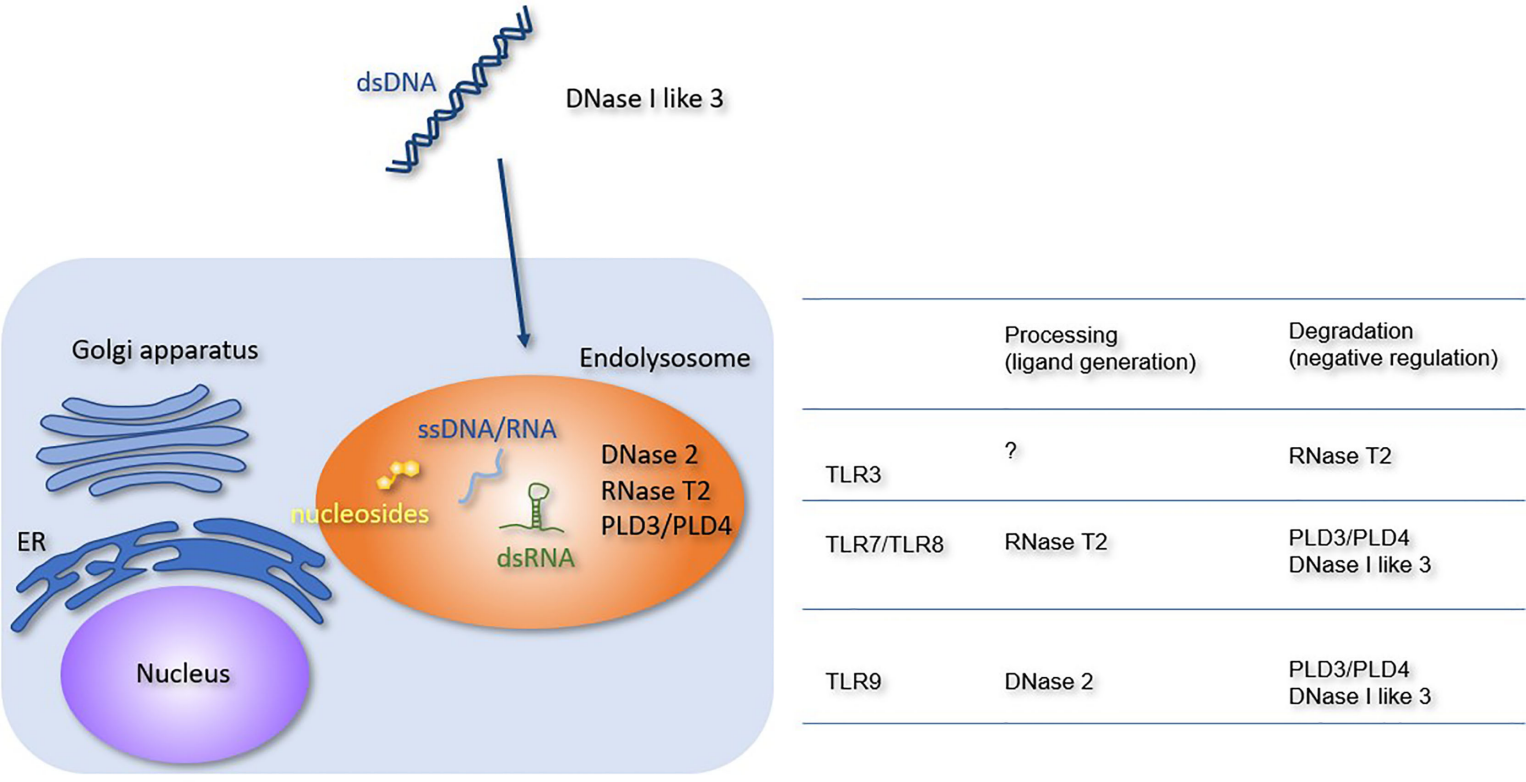
Processing or degradation of TLR ligands by DNases and RNases. The extracellular and lysosomal enzymes involved in NA metabolism are shown. The Table summarizes the role of each enzyme in TLR responses. RNase T2 negatively regulates TLR3 responses but is required for TLR7 and TLR8 responses. PLD3, PLD4, and DNase I-like 3 negatively regulate TLR7, TLR8, and TLR9 responses. DNase 2 is required for TLR9 response.

NAs released from dead cells are internalized into the endosomal compartment of the macrophages. Extracellular DNA is degraded by members of the DNASE1 family, such as DNase I and DNase I-like 3 (**Figure 1**). Because these enzymes require an optimal pH of 7.0, they degrade DNA before internalization into the endosomal compartment. DNase I is expressed in the kidney and lacrimal gland, whereas DNase I-like 3 is expressed in the innate immune cells, such as DCs. Despite their restricted expression, DNases are secreted and can degrade DNA in the circulation (16). Lupus-like diseases develop in patients harboring loss-of-function mutations in *DNASE1* or *DNASE1L3* genes (17, 18). Consistent with this, *Dnse1l3* deficiency in mice causes TLR7 and TLR9-dependent systemic autoimmune response (19). TLR7 activation in *Dnse1l3* ^−/−^ mice may be explained by TLR7 response to DNA-derived deoxyribonucleosides (2). Although DNase I is thought to negatively regulates TLR responses to self-DNA, TLR-dependency of lupus nephritis in *Dnase1* ^−/−^ mice has not been shown yet.

#### 1.2.2 DNase II

DNase II is expressed in various cell types and exhibits optimal activity at pH 5.0. It plays an indispensable role in DNA degradation in the endosomal compartment. Loss-of-function mutations in the *DNASE2* gene cause type I interferonopathy characterized by anemia, thrombocytopenia, hepatosplenomegaly, glomerulonephritis, and liver fibrosis (20). Consistent with this, *Dnase2a* ^−/−^ mice are embryonically lethal owing to type I IFN-dependent anemia (21). Type I IFN production is driven by the cGAS-STING axis, because *Dnase2a* ^−/−^ *Sting* ^−/−^ mice are born normal (22). Although DNA accumulates in the endosomal compartment, TLR9 is not involved in type II IFN-dependent lethality (23) because DNase II is required for generation of a TLR9 ligand in DCs (24). *Dnase2a* ^−/−^ mice rescued by type I IFN receptor deficiency suffer from arthritis due to the constitutive activation of cGAS-STING and another cytoplasmic dsDNA sensor absent in melanoma 2 (AIM2) (25). The activation of cytoplasmic dsDNA sensors in *Dnase2a* ^−/−^ mice raises the question of how lysosomal DNA enters the cytoplasm. dsDNAs are released from the nucleus to the cytoplasm under various stresses, and cytoplasmic dsDNAs are degraded by endosomal DNase II (26). cGAS-STING and AIM2 may be activated by nuclear DNA, which escapes lysosomal degradation in *Dnase2a* ^−/−^ mice.

#### 1.2.3 RNase T2

RNase T2, the member of the T2 family of RNases with optimal activity at pH 4–5, is broadly expressed in various cell types. RNase T2 degrades RNA in the endosomal compartment, such as ribosomal RNA (27, 28). Loss-of-function mutations in the *RNASET2* gene cause cystic leukoencephalopathy (29), and RNase T2-deficient mice show type I interferonopathy; however, the responsible RNA sensor remains unclear (30). RNase T2 negatively regulates TLR3 responses by degrading dsRNAs, whereas it is required for TLR7/8 responses *via* the generation of ligands (31–33). These RNA-sensing TLRs might play a role in cystic leukoencephalopathy.

#### 1.2.4 Phospholipase D3 and Phospholipase D4

Phospholipase D3 (PLD3) and Phospholipase D4 (PLD4) belong to the phospholipase D family. Macrophages express both PLD3 and PLD4, whereas B cells and DCs express only PLD4. Genome-wide association studies have shown that the *PLD4* gene is linked to autoimmune diseases, such as systemic sclerosis, systemic lupus erythematosus (SLE), and rheumatoid arthritis (34–36). In contrast, the *PLD3* gene is linked to neurodegenerative diseases, such as Alzheimer’s disease and spinocerebellar ataxia (37, 38). *Pld3^−/−^ Pld4^−/−^* mice exhibit macrophage activation syndrome (39, 40). PLD3 and PLD4 exonucleases degrade both DNA and RNA and negatively regulate TLR7 and TLR9 responses. The constitutive activation of TLR7 and TLR9 contributes to the pathology in *Pld3^−/−^ Pld4^−/−^* mice (39).

### 1.3 Endosomal Compartment as the Platform Controlling Endosomal TLRs

#### 1.3.1 Unc93B1

**Figure 2:**
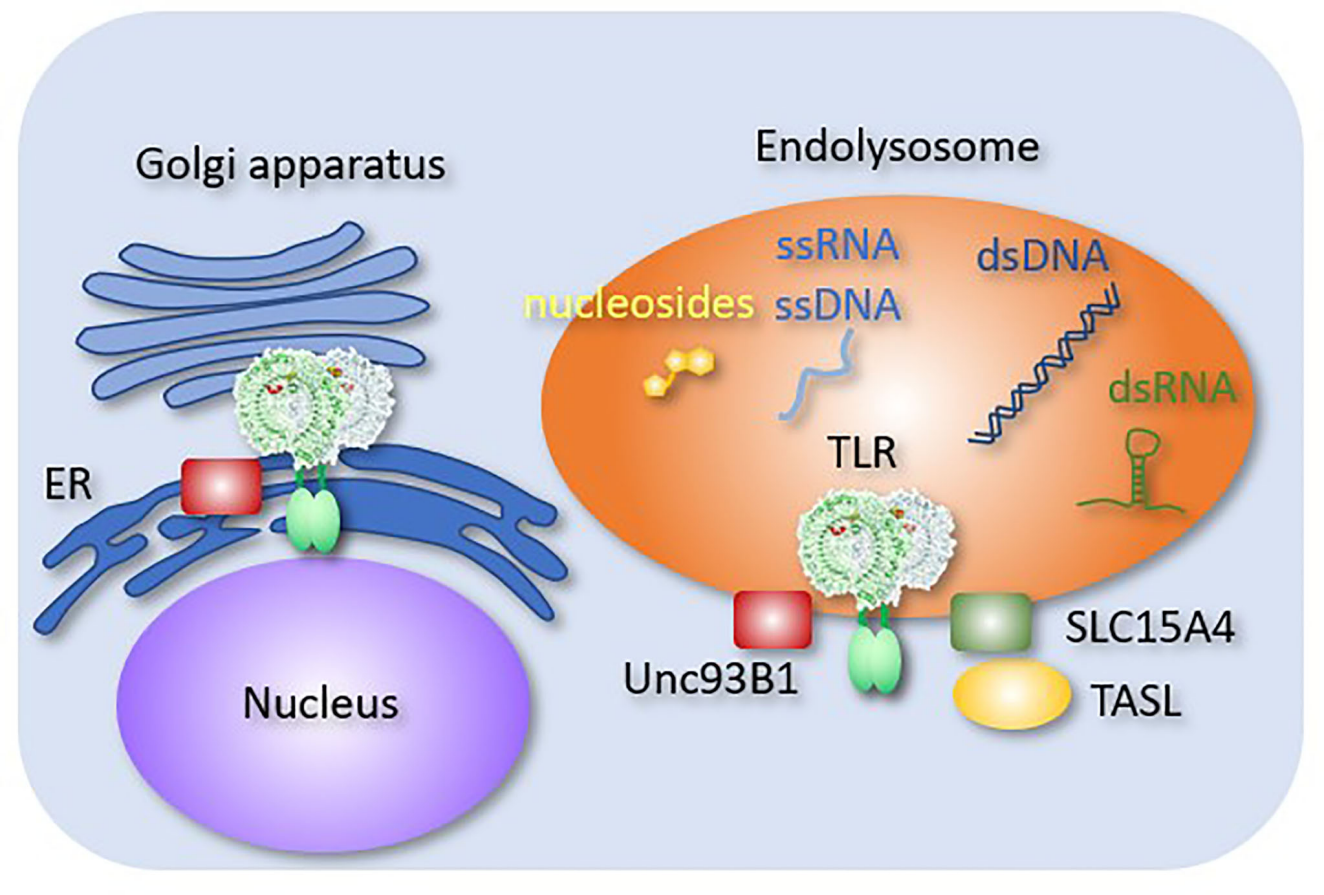
Endosomal molecules controlling TLR responses. Endosomal molecules that control TLR responses are shown. Unc93B1 negatively regulates TLR9 dimerization. A complex consisting of SLC15A4 and TASL mediates TLR-dependent type I IFN production.

Unc93B1 is a multi-transmembrane endoplasmic reticulum (ER) molecule that is directly associated with the endosomal TLRs, including TLR3, TLR5, TLR7, TLR8, TLR9, and TLR13 (**Figure 2**). Without Unc93B1, these TLRs remain in the ER and fail to respond to their cognate ligands (41). In addition to its role as a TLR-specific chaperone, Unc93B1 directly affects TLR response. For example, Unc93B1 dissociates from TLR9 and TLR3 upon ligand stimulation. If Unc93B1 stays with TLR9 and TLR3, these TLRs fail to dimerize and activate downstream signals (42, 43). In TLR7, Unc93B1 remains associated with ligated TLR7, but the complex is degraded after being transported into intralumenal vesicles (44). These results demonstrated the role of Unc93B1 as a negative regulator of endosomal TLR response by inhibiting dimerization or degradation. The D34A mutation of Un93B1 in mice causes systemic inflammation due to constitutive TLR7 activation (45), suggesting that Unc93B1 serves as a negative regulator of TLR7 at the steady state. However, little is known about the mechanism by which Unc93B1 dissociates from TLR9 or TLR3 upon ligand stimulation.

#### 1.3.2 Mechanisms of Type I IFN Production

TLR-dependent type I IFN production is controlled by the endosomal compartment in multiple ways. For instance, the endosomal compartment is the site where metabolic information is gathered. It is not surprising that metabolic sensors, such as mammalian target of rapamycin complex 1 (mTORC1), are localized in the endosomal compartment. Type I IFN production by TLR7 or TLR9 in pDCs is dependent on mTORC1 activation. Interestingly, proinflammatory cytokine production does not depend on mTORC1. Because mTORC1 activation drives anabolic processes in immune cells, type I IFN response might be more dependent on the anabolic activity than on proinflammatory responses.

TLR-dependent type I IFN induction is preceded by the stimulation of proinflammatory cytokines (46). This delayed type I IFN induction is ascribed to the requirement of endosomal trafficking for type I IFN production (47). Endosomal trafficking depends on small GTPases such as ADP ribosylation factors like 8b (Arl8b) and Rab7a. Interestingly, these proteins are differentially activated by TLRs. For example, TLR7 trafficking in pDCs depends on Arl8b, whereas TLR3 trafficking is regulated by Rab7a (46, 48). These GTPases mediate anterograde trafficking of TLR-containing endosomes from perinuclear regions to the cell periphery. Endosomal trafficking enables TLRs to interact with mTORC1 (49), suggesting that such trafficking connects the metabolic status with type I IFN responses.

TLR7 activation in pDCs initiates inside-out signaling of α_L_β_2_ integrin, the adhesion of which is required to induce endosomal trafficking (46). Consistent with this, cell-cell interactions enhance type I IFN production by pDCs (50). The initiation of type I IFN responses is likely to depend on the optimal environment, such as the anabolic process and cell adhesion. TLRs sense these environmental conditions through endosomal trafficking. In other words, environmental cues affect TLR-dependent type I IFN responses *via* endosomal positioning.

SLC15A3 and SLC15A4 are peptide transporters in endosomal compartments. These molecules transport endosomal muramyl dipeptides (MDPs), which are sensed by NOD2 in the cytoplasm (51). SLC15A4 is required for TLR7 and TLR9 responses in pDCs (52). It also mediates AP3-dependent endosomal trafficking required for TLR7 and TLR9 responses (53). Moreover, SLC15A4 serves as a scaffold protein by associating with TLR adaptor interacting with SLC15A4 on the lysosome (TASL) (54), which recruits IRFs to transmit signals from TLR7, TLR8, and TLR9. These molecules mediate the production of TLR-dependent type-I IFN. Interestingly, *SLC15A4*, *IRF5* and *TASL* are all lupus-associated genes (55), which strongly suggest that type I IFN production by endosomal TLRs is activated in SLE.

### 1.4 Inflammatory Diseases Associated With Dysregulated Responses of Endosomal TLRs

#### 1.4.1 Monogenic Diseases

Gain-of-function mutations in the *TLR8* gene such as P432L, F494L, and G527D, cause neutropenia, infections, lymphoproliferation, and B cell deficiency (56). Although TLR8 is expressed in myeloid cells, T cell activation and B cell deficiency develop, probably because of the cell non-autonomous mechanisms. These clinical manifestations are not necessarily consistent with the phenotypes of TLR8 transgenic mice, in which TLR8 expression is driven by a human endogenous promoter (57). TLR8 transgenic mice exhibit severe inflammation in the pancreas, salivary glands, and joints. In contrast to human patients harboring gain-of-function mutations in the *TLR8* gene, neither neutropenia nor B cell deficiency was observed. The TLR8 responses in TLR8 transgenic mice are distinct from those in humans.

Constitutive activation of TLR7 due to its gain-of-function mutation causes monogenic SLE in humans (58). The increase in B cell number depends on TLR7 expression. Because TLR7 is expressed not only in myeloid cells, but also in B cells, mutated TLR7 drives cell-autonomous B cell activation. A lupus-prone mouse strain, the Y-linked autoimmune accelerator strain, has an additional copy of the TLR7 gene that results in TLR7 hyperactivation, leading to lupus-like state (59, 60). Clinical manifestations in patients harboring gain-of-function mutations in *TLR7* genes differ from those with *TLR8* mutations and are ascribed to different expression in different immune cells. TLR7 is highly expressed in B cells and pDCs, whereas TLR8 is highly expressed in monocytes and macrophages.

The *ACP5* gene encodes lysosomal acid phosphatase expressed in osteoclasts, macrophages, and DCs. Loss-of-function mutations in the *ACP5* gene cause spondyloenchondrodysplasia with immune dysregulation, a disease characterized by skeletal dysplasia and neurologic and autoimmune manifestations (61). The detailed mechanisms underlying autoimmune manifestations remain unclear. ACP5 deficiency increases the level of hyperphosphorylated osteopontin, which is suggested to promote TLR9 responses in osteoclasts and macrophages.

#### 1.4.2 Polygenic Diseases

SLE is an autoimmune disease characterized by autoantibody production and clinical manifestations affecting the skin, joints, kidneys, and the central nervous system (62). Causative autoimmune responses are driven by autoreactive B cells that produce autoantibodies against NA-associated autoantigens and cDCs and pDCs that produce proinflammatory cytokines and type I IFN, respectively (63, 64). In addition to these cells, monocytes/macrophages infiltrate the glomeruli and play pathologic roles in glomerular damage associated with SLE, independent of immune complex (IC) deposition (65–67). The TLR7 agonist imiquimod drives lupus nephritis in mice (68, 69), whereas the pathologies in the lupus-prone strain, New Zealand Black/New Zealand White F1 (NZBWF1) mice, is ameliorated by TLR7 chemical inhibitor or by anti-TLR7 monoclonal antibody (70, 71). The number of Ly6C^low^ patrolling monocytes TLR7-dependently increases in NZBWF1 mice (39). Interestingly, during monocyte maturation from Ly6C^hi^ to Ly6C^low^ cells, TLR9 expression decreases with TLR7 expression unchanged (72). The IC-independent glomerular accumulation of Ly6C^low^ patrolling monocytes causes lupus nephritis in another lupus-prone mouse strain lacking the human SLE susceptibility gene, *Tnip1* (67).

The TLR7-dependent increase in Ly6C^low^ monocytes/macrophages might be driven by self-derived RNAs. The 60 kDa Ro60 ribonucleoprotein, also known as the SSA/Ro antigen, is one of the most studied autoantigens associated with SLE or primary Sjögren syndrome. Because Alu retroelements, repetitive transposons, bind to Ro60 and activate TLR7 and TLR8 (73), the IC consisting of Ro60, Alu retroelements, and autoantibodies is formed in lupus-prone mice and internalized by autoreactive B cells or DCs *via* the BCR or FcR, respectively. Alu retroelements in the IC activate endosomal TLR7 or TLR8 to drive autoimmunity (74). TLR7 may also be activated by RNA from bacteria, which enter the circulation through the leaky gut (75). Notably, commensal orthologs of Ro60 might play a pathologic role in SLE (76).

Systemic sclerosis (SSc) is a multisystem life-threatening fibrosing disorder (77). Aberrant TLR8 expression in pDCs has been reported in patients with SSc (78). pDCs normally express only TLR7. Additional expression of TLR8 may promote autoimmune responses in SSc.

## Author Contributions

KM wrote the manuscript. TS, RF, RS, SS, and YM made comments on the manuscript. All authors contributed to the article and approved the submitted version.

## Funding

This work was supported in part by: Grant-in-Aid for Scientific Research (S and A) to KM (16H06388, 21H04800), (B) to S-IS (26293083), and (C) (16K08827) to TS; JST CREST (JPMJCR13M5, JPMJCR21E4) to TS and KM, respectively; Joint Research Project of the Institute of Medical Science at the University of Tokyo; and JSPS KAKENHI Grant Number JP 16H06276 (AdAMS).

## Conflict of Interest

The authors declare that the research was conducted in the absence of any commercial or financial relationships that could be construed as potential conflicts of interest.

## Publisher’s Note

All claims expressed in this article are solely those of the authors and do not necessarily represent those of their affiliated organizations, or those of the publisher, the editors and the reviewers. Any product that may be evaluated in this article, or claim that may be made by its manufacturer, is not guaranteed or endorsed by the publisher.

## References

[1] Barton GM Kagan JC. A Cell Biological View of Toll-Like Receptor Function: Regulation Through Compartmentalization. Nat Rev Immunol (2009) 9(8):535–42. doi: 10.1038/nri2587 PMC3934928 19556980

[2] Shibata T Ohto U Nomura S Kibata K Motoi Y Zhang Y. Guanosine and its Modified Derivatives are Endogenous Ligands for TLR7. Int Immunol (2016) 28(5):211–22. doi: 10.1093/intimm/dxv062 PMC4888345 26489884

[3] Zhang Z Ohto U Shibata T Krayukhina E Taoka M Yamauchi Y. Structural Analysis Reveals That Toll-Like Receptor 7 Is a Dual Receptor for Guanosine and Single-Stranded RNA. Immunity (2016) 45(4):737–48. doi: 10.1016/j.immuni.2016.09.011 27742543

[4] Tanji H Ohto U Shibata T Taoka M Yamauchi Y Isobe T. Toll-Like Receptor 8 Senses Degradation Products of Single-Stranded RNA. Nat Struct Mol Biol (2015) 22(2):109–15. doi: 10.1038/nsmb.2943 25599397

[5] Miyake K Shibata T Ohto U Shimizu T Saitoh SI Fukui R. Mechanisms Controlling Nucleic Acid-Sensing Toll-Like Receptors. Int Immunol (2018) 30(2):43–51. doi: 10.1093/intimm/dxy016 29452403

[6] Kaisho T Akira S. Toll-Like Receptor Function and Signaling. J Allergy Clin Immunol (2006) 117(5):979–87. doi: 10.1016/j.jaci.2006.02.023 16675322

[7] Miyake K Saitoh SI Sato R Shibata T Fukui R Murakami Y. Endolysosomal Compartments as Platforms for Orchestrating Innate Immune and Metabolic Sensors. J Leukoc Biol (2019) 106(4):853–62. doi: 10.1002/JLB.MR0119-020R 31219657

[8] Bell JK Botos I Hall PR Askins J Shiloach J Segal DM. The Molecular Structure of the Toll-Like Receptor 3 Ligand-Binding Domain. Proc Natl Acad Sci U S A (2005) 102(31):10976–80. doi: 10.1073/pnas.0505077102 PMC1182468 16043704

[9] Zhang S-Y Casanova J-L. Inborn Errors Underlying Herpes Simplex Encephalitis: From TLR3 to IRF3. J Exp Med (2015) 212(9):1342. doi: 10.1084/jem.2129insight4 26304982 PMC4548049

[10] Bernard JJ Cowing-Zitron C Nakatsuji T Muehleisen B Muto J Borkowski AW. Ultraviolet Radiation Damages Self Noncoding RNA and Is Detected by TLR3. Nat Med (2012) 18:1286–90. doi: 10.1038/nm.2861. PMC3812946 22772463

[11] Takemura N Kawasaki T Kunisawa J Sato S Lamichhane A Kobiyama K. Blockade of TLR3 Protects Mice From Lethal Radiation-Induced Gastrointestinal Syndrome. Nat Commun (2014) 5:3492. doi: 10.1038/ncomms4492 24637670 PMC3959210

[12] Song W Wang J Han Z Zhang Y Zhang H Wang W. Structural Basis for Specific Recognition of Single-Stranded RNA by Toll-Like Receptor 13. Nat Struct Mol Biol (2015) 22(10):782–7. doi: 10.1038/nsmb.3080 26323037

[13] Oldenburg M Krüger A Ferstl R Kaufmann A Nees G Sigmund A. TLR13 Recognizes Bacterial 23S rRNA Devoid of Erythromycin Resistance-Forming Modification. Science (2012) 337(6098):1111–5. doi: 10.1126/science.1220363 22821982

[14] Ohto U Shibata T Tanji H Ishida H Krayukhina E Uchiyama S. Structural Basis of CpG and Inhibitory DNA Recognition by Toll-Like Receptor 9. Nature (2015) 520(7549):702–5. doi: 10.1038/nature14138 25686612

[15] Mouchess ML Arpaia N Souza G Barbalat R Ewald SE Lau L. Transmembrane Mutations in Toll-Like Receptor 9 Bypass the Requirement for Ectodomain Proteolysis and Induce Fatal Inflammation. Immunity (2011) 35(5):721–32. doi: 10.1016/j.immuni.2011.10.009 PMC3230302 22078797

[16] Jiménez-Alcázar M Rangaswamy C Panda R Bitterling J Simsek YJ Long AT. Host DNases Prevent Vascular Occlusion by Neutrophil Extracellular Traps. Science (2017) 358(6367):1202–6. doi: 10.1126/science.aam8897 29191910

[17] Yasutomo K Horiuchi T Kagami S Tsukamoto H Hashimura C Urushihara M. Mutation of DNASE1 in People With Systemic Lupus Erythematosus. Nat Genet (2001) 28(4):313–4. doi: 10.1038/91070 11479590

[18] Al-Mayouf SM Sunker A Abdwani R Abrawi SA Almurshedi F Alhashmi N. Loss-Of-Function Variant in DNASE1L3 Causes a Familial Form of Systemic Lupus Erythematosus. Nat Genet (2011) 43(12):1186–8. doi: 10.1038/ng.975 22019780

[19] Soni C Perez OA Voss WN Pucella JN Serpas L Mehl J. Plasmacytoid Dendritic Cells and Type I Interferon Promote Extrafollicular B Cell Responses to Extracellular Self-DNA. Immunity (2020) 52(6):1022–38.e7. doi: 10.1016/j.immuni.2020.04.015 32454024 PMC7306002

[20] Rodero MP Tesser A Bartok E Rice GI Della Mina E Depp M. Type I Interferon-Mediated Autoinflammation Due to DNase II Deficiency. Nat Commun (2017) 8(1):2176. doi: 10.1038/s41467-017-01932-3 29259162 PMC5736616

[21] Yoshida H Okabe Y Kawane K Fukuyama H Nagata S. Lethal Anemia Caused by Interferon-Beta Produced in Mouse Embryos Carrying Undigested DNA. Nat Immunol (2005) 6(1):49–56. doi: 10.1038/ni1146 15568025

[22] Ahn J Gutman D Saijo S Barber GN. STING Manifests Self DNA-Dependent Inflammatory Disease. Proc Natl Acad Sci U S A (2012) 109(47):19386–91. doi: 10.1073/pnas.1215006109 PMC3511090 23132945

[23] Okabe Y Kawane K Akira S Taniguchi T Nagata S. Toll-Like Receptor-Independent Gene Induction Program Activated by Mammalian DNA Escaped From Apoptotic DNA Degradation. J Exp Med (2005) 202(10):1333–9. doi: 10.1084/jem.20051654 PMC2212973 16301743

[24] Chan MP Onji M Fukui R Kawane K Shibata T Saitoh S. DNase II-Dependent DNA Digestion Is Required for DNA Sensing by TLR9. Nat Commun (2015) 6:5853. doi: 10.1038/ncomms6853 25600358

[25] Baum R Sharma S Carpenter S Li Q-Z Busto P Fitzgerald KA. Cutting Edge: AIM2 and Endosomal TLRs Differentially Regulate Arthritis and Autoantibody Production in DNase II–Deficient Mice. J Immunol (2015) 194(3):873–7. doi: 10.4049/jimmunol.1402573 PMC4299698 25548216

[26] Lan Yuk Y Londoño D Bouley R Rooney Michael S Hacohen N. Dnase2a Deficiency Uncovers Lysosomal Clearance of Damaged Nuclear DNA *via* Autophagy. Cell Rep (2014) 9(1):180–92. doi: 10.1016/j.celrep.2014.08.074 PMC4555847 25284779

[27] Huang H Kawamata T Horie T Tsugawa H Nakayama Y Ohsumi Y. Bulk RNA Degradation by Nitrogen Starvation-Induced Autophagy in Yeast. EMBO J (2015) 34(2):154–68. doi: 10.15252/embj.201489083 PMC4337068 25468960

[28] Haud N Kara F Diekmann S Henneke M Willer JR Hillwig MS. Rnaset2 Mutant Zebrafish Model Familial Cystic Leukoencephalopathy and Reveal a Role for RNase T2 in Degrading Ribosomal RNA. Proc Natl Acad Sci U S A (2011) 108(3):1099–103. doi: 10.1073/pnas.1009811107 PMC3024650 21199949

[29] Henneke M Diekmann S Ohlenbusch A Kaiser J Engelbrecht V Kohlschutter A. RNASET2-Deficient Cystic Leukoencephalopathy Resembles Congenital Cytomegalovirus Brain Infection. Nat Genet (2009) 41(7):773–5. doi: 10.1038/ng.398 19525954

[30] Kettwig M Ternka K Wendland K Krüger DM Zampar S Schob C. Interferon-Driven Brain Phenotype in a Mouse Model of RNaseT2 Deficient Leukoencephalopathy. Nat Commun (2021) 12(1):6530. doi: 10.1038/s41467-021-26880-x 34764281 PMC8586222

[31] Ostendorf T Zillinger T Andryka K Schlee-Guimaraes TM Schmitz S Marx S. Immune Sensing of Synthetic, Bacterial, and Protozoan RNA by Toll-Like Receptor 8 Requires Coordinated Processing by RNase T2 and RNase 2. Immunity (2020) 52(4):591–605. doi: 10.1016/j.immuni.2020.03.009 32294405

[32] Greulich W Wagner M Gaidt MM Stafford C Cheng Y Linder A. TLR8 Is a Sensor of RNase T2 Degradation Products. Cell (2019) 179(6):1264–75.e13. doi: 10.1016/j.cell.2019.11.001 31778653 PMC7116005

[33] Liu K Sato R Shibata T Hiranuma R Reuter T Fukui R. Skewed Endosomal RNA Responses From TLR7 to TLR3 in RNase T2-Deficient Macrophages. Int Immunol (2021) 33(9):479–90. doi: 10.1093/intimm/dxab033 34161582

[34] Akizuki S Ishigaki K Kochi Y Law SM Matsuo K Ohmura K. PLD4 is a Genetic Determinant to Systemic Lupus Erythematosus and Involved in Murine Autoimmune Phenotypes. Ann Rheum Dis (2019) 78(4):509–18. doi: 10.1136/annrheumdis-2018-214116 30679154

[35] Terao C Ohmura K Kawaguchi Y Nishimoto T Kawasaki A Takehara K. PLD4 as a Novel Susceptibility Gene for Systemic Sclerosis in a Japanese Population. Arthritis Rheumatol (2013) 65(2):472–80. doi: 10.1002/art.37777 23124809

[36] Okada Y Terao C Ikari K Kochi Y Ohmura K Suzuki A. Meta-Analysis Identifies Nine New Loci Associated With Rheumatoid Arthritis in the Japanese Population. Nat Genet (2012) 44(5):511–6. doi: 10.1038/ng.2231 22446963

[37] Cruchaga C Karch CM Jin SC Benitez BA Cai Y Guerreiro R. Rare Coding Variants in the Phospholipase D3 Gene Confer Risk for Alzheimer's Disease. Nature (2014) 505(7484):550–4. doi: 10.1038/nature12825 PMC4050701 24336208

[38] Nibbeling EAR Duarri A Verschuuren-Bemelmans CC Fokkens MR Karjalainen JM Smeets CJLM. Exome Sequencing and Network Analysis Identifies Shared Mechanisms Underlying Spinocerebellar Ataxia. Brain (2017) 140(11):2860–78. doi: 10.1093/brain/awx251 29053796

[39] Gavin AL Huang D Blane TR Thinnes TC Murakami Y Fukui R. Cleavage of DNA and RNA by PLD3 and PLD4 Limits Autoinflammatory Triggering by Multiple Sensors. Nat Commun (2021) 12(1):5874. doi: 10.1038/s41467-021-26150-w 34620855 PMC8497607

[40] Gavin AL Huang D Huber C Mårtensson A Tardif V Skog PD. PLD3 and PLD4 are Single-Stranded Acid Exonucleases That Regulate Endosomal Nucleic-Acid Sensing. Nat Immunol (2018) 19(9):942–53. doi: 10.1038/s41590-018-0179-y PMC6105523 30111894

[41] Kim YM Brinkmann MM Paquet ME Ploegh HL. UNC93B1 Delivers Nucleotide-Sensing Toll-Like Receptors to Endolysosomes. Nature (2008) 452(7184):234–8. doi: 10.1038/nature06726 18305481

[42] Majer O Liu B Woo BJ Kreuk LSM Van Dis E Barton GM. Release From UNC93B1 Reinforces the Compartmentalized Activation of Select TLRs. Nature (2019) 575(7782):371–4. doi: 10.1038/s41586-019-1611-7 PMC6856438 31546247

[43] Ishida H Asami J Zhang Z Nishizawa T Shigematsu H Ohto U. Cryo-EM Structures of Toll-Like Receptors in Complex With UNC93B1. Nat Struct Mol Biol (2021) 28(2):173–80. doi: 10.1038/s41594-020-00542-w 33432245

[44] Majer O Liu B Kreuk LSM Krogan N Barton GM. UNC93B1 Recruits Syntenin-1 to Dampen TLR7 Signalling and Prevent Autoimmunity. Nature (2019) 575(7782):366–70. doi: 10.1038/s41586-019-1612-6 PMC6856441 31546246

[45] Fukui R Saitoh S Kanno A Onji M Shibata T Ito A. Unc93B1 Restricts Systemic Lethal Inflammation by Orchestrating Toll-Like Receptor 7 and 9 Trafficking. Immunity (2011) 35(1):69–81. doi: 10.1016/j.immuni.2011.05.010 21683627

[46] Saitoh SI Abe F Kanno A Tanimura N Mori Saitoh Y Fukui R. TLR7 Mediated Viral Recognition Results in Focal Type I Interferon Secretion by Dendritic Cells. Nat Commun (2017) 8(1):1592. doi: 10.1038/s41467-017-01687-x 29150602 PMC5693993

[47] Cao W Manicassamy S Tang H Kasturi SP Pirani A Murthy N. Toll-Like Receptor-Mediated Induction of Type I Interferon in Plasmacytoid Dendritic Cells Requires the Rapamycin-Sensitive PI(3)K-mTOR-P70s6k Pathway. Nat Immunol (2008) 9(10):1157–64. doi: 10.1038/ni.1645 PMC3732485 18758466

[48] Sato R Kato A Chimura T Saitoh SI Shibata T Murakami Y. Combating Herpesvirus Encephalitis by Potentiating a TLR3-Mtorc2 Axis. Nat Immunol (2018) 19(10):1071–82. doi: 10.1038/s41590-018-0203-2 30201994

[49] Korolchuk VI Saiki S Lichtenberg M Siddiqi FH Roberts EA Imarisio S. Lysosomal Positioning Coordinates Cellular Nutrient Responses. Nat Cell Biol (2011) 13(4):453–60. doi: 10.1038/ncb2204 PMC3071334 21394080

[50] Kim S Kaiser V Beier E Bechheim M Guenthner-Biller M Ablasser A. Self-Priming Determines High Type I IFN Production by Plasmacytoid Dendritic Cells. Eur J Immunol (2014) 44(3):807–18. doi: 10.1002/eji.201343806 PMC4523003 24338737

[51] Nakamura N Lill JR Phung Q Jiang Z Bakalarski C de Mazière A. Endosomes are Specialized Platforms for Bacterial Sensing and NOD2 Signalling. Nature (2014) 509(7499):240–4. doi: 10.1038/nature13133 24695226

[52] Kobayashi T Shimabukuro-Demoto S Yoshida-Sugitani R Furuyama-Tanaka K Karyu H Sugiura Y. The Histidine Transporter SLC15A4 Coordinates mTOR-Dependent Inflammatory Responses and Pathogenic Antibody Production. Immunity (2014) 41(3):375–88. doi: 10.1016/j.immuni.2014.08.011 25238095

[53] Rimann I Gonzalez-Quintial R Baccala R Kiosses WB Teijaro JR Parker CG. The Solute Carrier SLC15A4 Is Required for Optimal Trafficking of Nucleic Acid-Sensing TLRs and Ligands to Endolysosomes. Proc Natl Acad Sci U S A (2022) 119(14):e2200544119. doi: 10.1073/pnas.2200544119 35349343 PMC9169117

[54] Heinz LX Lee J Kapoor U Kartnig F Sedlyarov V Papakostas K. TASL is the SLC15A4-Associated Adaptor for IRF5 Activation by TLR7-9. Nature (2020) 581(7808):316–22. doi: 10.1038/s41586-020-2282-0 PMC7610944 32433612

[55] Bentham J Morris DL Graham DSC Pinder CL Tombleson P Behrens TW. Genetic Association Analyses Implicate Aberrant Regulation of Innate and Adaptive Immunity Genes in the Pathogenesis of Systemic Lupus Erythematosus. Nat Genet (2015) 47(12):1457–64. doi: 10.1038/ng.3434 PMC4668589 26502338

[56] Aluri J Bach A Kaviany S Chiquetto Paracatu L Kitcharoensakkul M Walkiewicz MA. Immunodeficiency and Bone Marrow Failure With Mosaic and Germline TLR8 Gain of Function. Blood (2021) 137(18):2450–62. doi: 10.1182/blood.2020009620 PMC8109013 33512449

[57] Guiducci C Gong M Cepika AM Xu Z Tripodo C Bennett L. RNA Recognition by Human TLR8 can Lead to Autoimmune Inflammation. J Exp Med (2013) 210(13):2903–19. doi: 10.1084/jem.20131044 PMC3865472 24277153

[58] Brown GJ Cañete PF Wang H Medhavy A Bones J Roco JA. TLR7 Gain-of-Function Genetic Variation Causes Human Lupus. Nature (2022) 605:349–56. doi: 10.1038/s41586-022-04642-z PMC9095492 35477763

[59] Pisitkun P Deane JA Difilippantonio MJ Tarasenko T Satterthwaite AB Bolland S. Autoreactive B Cell Responses to RNA-Related Antigens Due to TLR7 Gene Duplication. Science (2006) 312(5780):1669. doi: 10.1126/science.1124978 16709748

[60] Subramanian S Tus K Li Q-Z Wang A Tian X-H Zhou J. A Tlr7 Translocation Accelerates Systemic Autoimmunity in Murine Lupus. Proc Natl Acad Sci USA (2006) 103(26):9970–5. doi: 10.1073/pnas.0603912103 PMC1502563 16777955

[61] Briggs TA Rice GI Daly S Urquhart J Gornall H Bader-Meunier B. Tartrate-Resistant Acid Phosphatase Deficiency Causes a Bone Dysplasia With Autoimmunity and a Type I Interferon Expression Signature. Nat Genet (2011) 43(2):127–31. doi: 10.1038/ng.748 PMC3030921 21217755

[62] Yu F Haas M Glassock R Zhao MH. Redefining Lupus Nephritis: Clinical Implications of Pathophysiologic Subtypes. Nat Rev Nephrol (2017) 13(8):483–95. doi: 10.1038/nrneph.2017.85 28669995

[63] Smith CK Kaplan MJ. The Role of Neutrophils in the Pathogenesis of Systemic Lupus Erythematosus. Curr Opin Rheumatol (2015) 27(5):448–53. doi: 10.1097/BOR.0000000000000197 PMC12351535 26125102

[64] Tsokos GC Lo MS Reis PC Sullivan KE. New Insights Into the Immunopathogenesis of Systemic Lupus Erythematosus [Review]. Nat Rev Rheumatol (2016) 12(12):716–30. doi: 10.1038/nrrheum.2016.186 27872476

[65] Clynes R Dumitru C Ravetch JV. Uncoupling of Immune Complex Formation and Kidney Damage in Autoimmune Glomerulonephritis. Science (1998) 279(5353):1052–4. doi: 10.1126/science.279.5353.1052 9461440

[66] Bergtold A Gavhane A D'Agati V Madaio M Clynes R. FcR-Bearing Myeloid Cells are Responsible for Triggering Murine Lupus Nephritis. J Immunol (2006) 177(10):7287–95. doi: 10.4049/jimmunol.177.10.7287 17082647

[67] Kuriakose J Redecke V Guy C Zhou J Wu R Ippagunta SK. Patrolling Monocytes Promote the Pathogenesis of Early Lupus-Like Glomerulonephritis. J Clin Invest (2019) 129(6):2251–65. doi: 10.1172/JCI125116 PMC6546471 31033479

[68] Goel RR Wang X O'Neil LJ Nakabo S Hasneen K Gupta S. Interferon Lambda Promotes Immune Dysregulation and Tissue Inflammation in TLR7-Induced Lupus. Proc Natl Acad Sci U S A (2020) 117(10):5409–19. doi: 10.1073/pnas.1916897117 PMC7071891 32094169

[69] Yokogawa M Takaishi M Nakajima K Kamijima R Fujimoto C Kataoka S. Epicutaneous Application of Toll-Like Receptor 7 Agonists Leads to Systemic Autoimmunity in Wild-Type Mice: A New Model of Systemic Lupus Erythematosus. Arthritis Rheumatol (2014) 66(3):694–706. doi: 10.1002/art.38298 24574230

[70] Tojo S Zhang Z Matsui H Tahara M Ikeguchi M Kochi M. Structural Analysis Reveals TLR7 Dynamics Underlying Antagonism. Nat Commun (2020) 11(1):5204. doi: 10.1038/s41467-020-19025-z 33060576 PMC7562955

[71] Murakami Y Fukui R Tanaka R Motoi Y Kanno A Sato R. Anti-TLR7 Antibody Protects Against Lupus Nephritis in NZBWF1 Mice by Targeting B Cells and Patrolling Monocytes. Front Immunol (2021) 12:777197. doi: 10.3389/fimmu.2021.777197 34868046 PMC8632649

[72] Sato R Reuter T Hiranuma R Shibata T Fukui R Motoi Y. The Impact of Cell Maturation and Tissue Microenvironments on the Expression of Endosomal Toll-Like Receptors in Monocytes and Macrophages. Int Immunol (2020) 32(12):785–98. doi: 10.1093/intimm/dxaa055 32840578

[73] Hung T Pratt GA Sundararaman B Townsend MJ Chaivorapol C Bhangale T. The Ro60 Autoantigen Binds Endogenous Retroelements and Regulates Inflammatory Gene Expression. Science (2015) 350(6259):455. doi: 10.1126/science.aac7442 26382853 PMC4691329

[74] Marshak-Rothstein A Rifkin IR. Immunologically Active Autoantigens: The Role of Toll-Like Receptors in the Development of Chronic Inflammatory Disease. Annu Rev Immunol (2007) 25:419–41. doi: 10.1146/annurev.immunol.22.012703.104514 17378763

[75] Manfredo Vieira S Hiltensperger M Kumar V Zegarra-Ruiz D Dehner C Khan N. Translocation of a Gut Pathobiont Drives Autoimmunity in Mice and Humans. Science (2018) 359(6380):1156–61. doi: 10.1126/science.aar7201 PMC5959731 29590047

[76] Greiling TM Dehner C Chen X Hughes K Iñiguez AJ Boccitto M. Commensal Orthologs of the Human Autoantigen Ro60 as Triggers of Autoimmunity in Lupus. Sci Transl Med (2018) 10(434):eaan2306. doi: 10.1126/scitranslmed.aan2306 29593104 PMC5918293

[77] Varga J Abraham D. Systemic Sclerosis: A Prototypic Multisystem Fibrotic Disorder. J Clin Invest (2007) 117(3):557–67. doi: 10.1172/JCI31139 PMC1804347 17332883

[78] Ah Kioon MD Tripodo C Fernandez D Kirou KA Spiera RF Crow MK. Plasmacytoid Dendritic Cells Promote Systemic Sclerosis With a Key Role for TLR8. Sci Transl Med (2018) 10(423):01. doi: 10.1126/scitranslmed.aam8458 PMC9865429 29321259

